# Synthesis and Chemical and Biological Evaluation of a Glycine Tripeptide Chelate of Magnesium

**DOI:** 10.3390/molecules26092419

**Published:** 2021-04-21

**Authors:** Derek R. Case, Jon Zubieta, Ren Gonzalez, Robert P. Doyle

**Affiliations:** 1 Department of Chemistry, 111 College Place, Syracuse University, Syracuse, NY 13244, USA; drcase@syr.edu (D.R.C.); jazubiet@syr.edu (J.Z.); 2 Balchem Corporation, 52 Sunrise Park Road, New Hampton, NY 10958, USA; ren.gonzalez@Balchem.com

**Keywords:** magnesium, triglycine, amino acid chelate, solubility, CaCo-2 cells

## Abstract

Magnesium (Mg^2+^) plays a crucial role in over 80% of all metabolic functions. It is becoming increasingly apparent that magnesium deficiency (hypomagnesemia) may play an important role in chronic disease. To counteract magnesium deficiency, there is an unmet clinical need to develop new fully characterized, highly bioavailable, and substantially water-soluble magnesium supplements. To this end, triglycine (HG_3_), a tripeptide of the amino acid glycine, was chosen as a chelating ligand for magnesium, given its natural occurrence and water solubility, and entropically-driven metal binding. Herein, we discuss the synthesis, chemical and physical characterization, and cellular uptake of a magnesium triglycine chelate (MgG_3_), an octahedral complex with extraordinary water solubility and improved cellular uptake in CaCo-2 cells than select commonly used magnesium supplements.

## 1. Introduction

As the 11th most abundant mineral in the human body, magnesium (Mg^2+^) is a critical micronutrient [1,2,3]. Magnesium is implicated in over 80% of metabolic functions and presides over 350 enzymatic processes [1,2,3]. Of total body Mg^2+^, ~99% is intracellular, with approximately 90% compartmentalized in bone and muscle, and the remainder (<1%) being distributed in plasma and erythrocytes. [4] This distribution of Mg^2+^ results in blood magnesium concentration being an inaccurate determinant of total body magnesium [5,6]. Subsequently, most Mg^2+^ deficiency (hypomagnesemia—defined as ˂0.75 mmol/L) [7] goes undiagnosed [8,9,10,11,12].

The prevalence of hypomagnesemia is due, in part, to processed foods having a significantly lowered magnesium content relative to natural crops and grains [13], and an increase in the consumption of such processed foods in the western world [14]. Hypomagnesemia has been linked to a litany of chronic medical conditions including hypertension [15], migraines [16], diabetes mellitus [17], cardiovascular disease [18], and osteoporosis [19,20,21].

Multiple studies support magnesium supplementation as a viable and generally safe means to treat hypomagnesemia [21,22,23,24]. Typical magnesium supplements include magnesium oxide, magnesium chloride, magnesium sulfate, and other organic magnesium formulations (see Table 1). Aqueous solubilities of such supplements vary greatly, from poorly soluble magnesium oxide to more soluble magnesium salts (Table 1).

There is a corollary between the solubility of magnesium compounds and their subsequent bioavailability—the more soluble the supplement, typically, the greater the bioavailability [34,35,36]. As such, although magnesium oxide is commonly used as a magnesium supplement for its ~60% magnesium composition, it lacks substantial water solubility and exhibits lower bioavailability than other organic magnesium salts [34,36]. While the chloride and sulfate magnesium salts exhibit substantial water solubility, they rapidly dissociate and leave available reactive sites on the Mg^2+^ site, making it more susceptible to precipitating from bioagents, such as phytates, and hydration—thus resulting in laxation [3,37,38,39]. As such, biologically relevant chelate ligands are utilized to occupy reactive sites on the magnesium to reduce laxation and increase solubility, subsequently resulting in greater bioavailability. This bioavailability is highly significant, as it will determine magnesium uptake within the body, and is largely dependent on a relatively acidic pH, as magnesium is taken up unequally throughout the length of the small intestine [37,38,40].

The naturally occurring glycine peptide trimer, triglycine (IUPAC: 2-[[2-[(2aminoacetyl)amino]acetyl]amino]acetic acid—HG_3_: not to be confused with trisglycine, three individual glycine monomers bound to a metal ion) [41], isolated from the Ackee (*Blighia sapida*) seed in 1968 by Fowden et al. [42], was chosen as the magnesium chelate ligand by virtue of several significant characteristics, namely, multiple Lewis base moieties that give rise to entropically favored binding (bidentate, tridentate, or tetradentate), extensive hydrogen bond donors/acceptors—when both free and complexed—offering inherent stability [43], well-understood coordination chemistry through the carboxylic acid moiety [44], weakly acidic character [37]; and evidence indicating a higher achievable plasma concentration than both its monomer and dimer progenitors [45].

Synthesis of a magnesium-triglycine chelate was carried out in water, and the recovered solid was analyzed via thermogravimetric analysis (TGA), differential scanning calorimetry (DSC), elemental analysis (EA), Infrared radiation spectroscopy (FT-IR), 1D- and 2D-nuclear magnetic resonance spectroscopy (NMR), and electrospray ionization mass spectrometry (ESI-MS). Cellular uptake of the magnesium triglycine complex was evaluated in the Human CaCo-2 cell line, as a model for the lower intestine [46]—where the majority of magnesium occurs [1]. Both solid and solution state analyses indicate the successful synthesis of a 6-coordinate octahedral magnesium complex via tetradentate HG_3_ employing a N_3_O donor set and two bound water molecules. Subsequent charge balance is achieved via a hydroxy anion. Solubility studies show that the MgG_3_ complex shows substantially increased water solubility at pH = 7 (169 ± 12.5 g/100 mL) relative to both magnesium salts and other current magnesium nutraceuticals. Cellular uptake studies in a CaCo-2 cell line indicated that MgG_3_ also shows greater relative total magnesium uptake than magnesium chloride and magnesium bisglycinate, suggesting MgG_3_ is an excellent lead for novel therapeutic magnesium compounds of this type.

## 2. Results

### 2.1. Characterization of MgG_3_

#### 2.1.1. Electrospray Ionization Mass Spectroscopy

**Figure 1:**
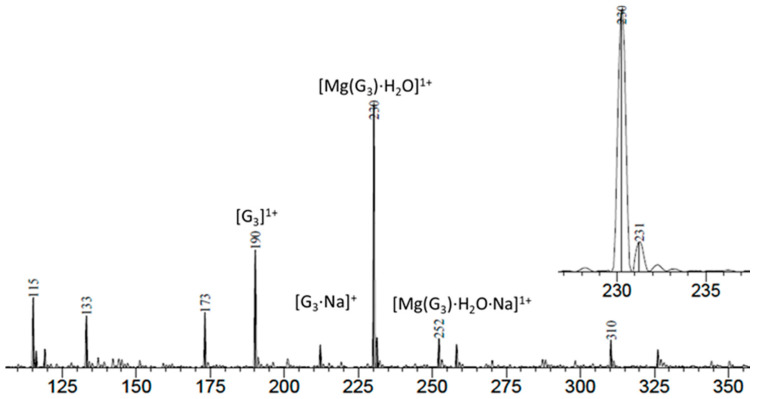
ESI-MS of MgG_3_ indicating the presence of both the free HG_3_ ligand as well as the chelate HG_3_ ligand. Inset at right exhibits the appropriate isotopic distribution pattern of a magnesium chelate.

To determine the presence of the desired product from the synthesis of MgG_3_, a mass spectrum of the dried MgG_3_ resuspended in 18 MΩ H_2_O indicated the presence of the free [HG_3_]^1+^ ligand, and the subsequent sodium adduct [HG_3_·Na]^+^ predicted at 190 *m/z* and 212 *m/z*, respectively, as well as presence of the monoaqua triglycine magnesium chelate [MgG_3_ + H_2_O]^+^ and the subsequent sodium adduct [MgG_3_ + H_2_O + Na]^+^ at 230 *m/z* and 252 *m/z*, respectively, as indicated by Figure 1.

#### 2.1.2. Structural Characterization of MgG_3_ via ^1^H-NMR and 2D ^1^H/^13^C HSQC/HMBC NMR

**Figure 2:**
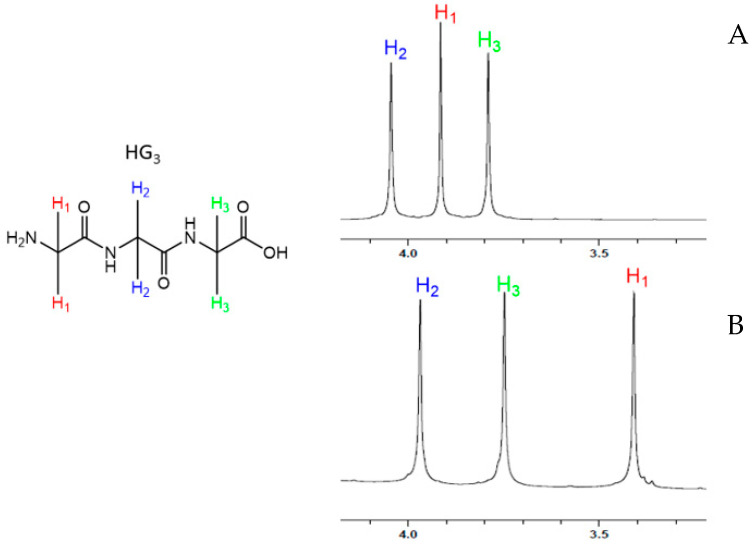
Proton NMR overlay of HG_3_ (**A**) and MgG_3_ (**B**) showing the observable upfield shifts of all protons and the subsequent change in proton splitting pattern. Full spectra in Supplemental Figure S7.

It is well established, in the literature, that divalent metal amino acid chelates exhibit ligand chelation predominantly through the carboxylic acid hydroxyl group and the nearest amine (e.g., glycine and glutamate) [43,44], but entropically-favored tri- and tetradentate complexes will occur primarily (e.g., aspartate) [44]. Ligand chelation to magnesium typically results in an observable shift of the proton signals adjacent to the Lewis bases of the free ligand that participate in coordination, as a consequence of the electropositive character of the metal [47,48,49]. Subsequently, ^1^H-NMR was conducted on both the free HG_3_ ligand and purified MgG_3_ in 700 μL of H_2_O/D_2_O (1:6 (*v:v*)). The uncomplexed HG_3_ exhibited three singlets at 4.044, 3.914 and 3.786 ppm, whereas the MgG_3_ complex showed an observable shift for each of the proton peaks at 3.967, 3.747, 3.4077, respectively (Figure S6), indicating a confirmed upfield shift of 0.5 ppm for H_1_ and 0.1 ppm for H_2_, and a tentative shift of 0.04 ppm assigned to H_3_ within the realm of experimental error (Figure 2)**.**

**Figure 3:**
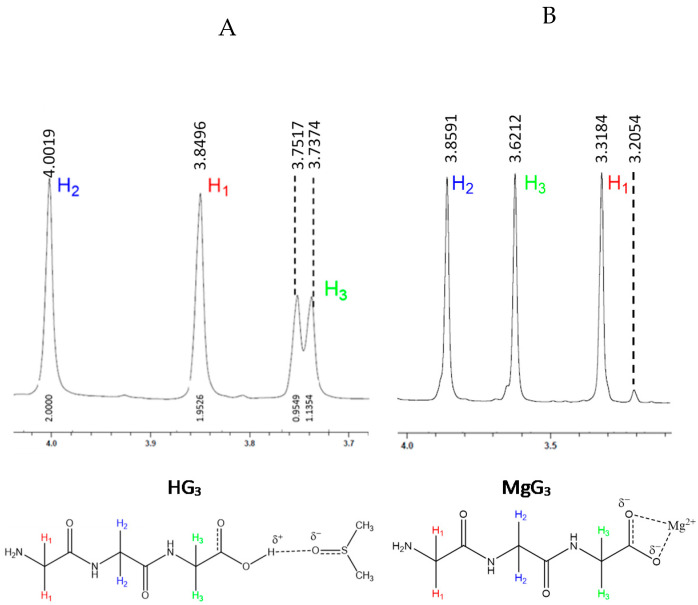
^1^H-NMR of HG_3_ in DMSO-d_6_ (**A**) showing an observable split (J = 5.7 Hz) of the carboxylic acid proton due to participation in hydrogen bonding, and the ^1^H-NMR of MgG_3_ (**B**) no longer showing the observable split of the carboxylic acid proton, suggesting that the ligand has coordinated to the magnesium through the carboxylic acid. Full spectra available in Supplementary Figure S9.

With the understanding that the HG_3_ ligand will coordinate through the carboxylic acid, subsequent ^1^H-NMR in a solution of 700 μL of DMSO-d_6_/H_2_O (1:6 (*v:v*)) was conducted to determine the coordinative participation of this moiety. It was hypothesized that the free proton of the carboxylic acid, when in the presence of the polar aprotic solvent DMSO, would participate in hydrogen bonding, thus resulting in an observable splitting of the H_3_ proton signal; when coordinated, the HG_3_ ligand would lose this carboxylic proton, thus eliminating the presence of an observable proton split. Subsequent ^1^H-NMR supported this hypothesis, thus implicating the carboxylic acid moiety as a participant in coordination to the magnesium metal (Figure 3).

**Figure 4:**
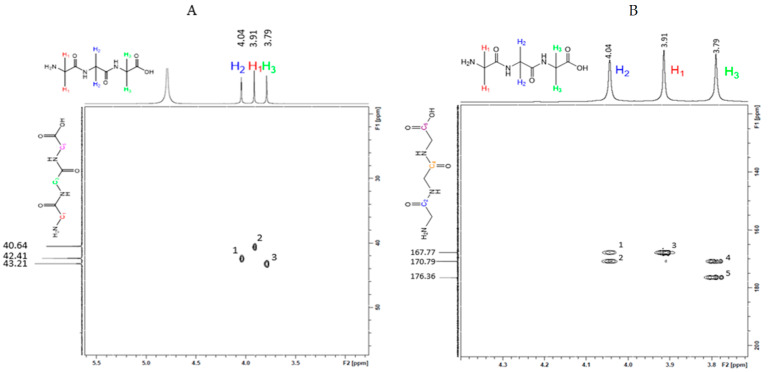
2D HSQC of HG_3_ (**A**) showing a single coherence point between each proton and its corresponding carbon (1:1:1) and the 2D HMBC of HG_3_ (**B**) showing two correlation points between H_2_ and two carbons (C_2_–167.8 ppm; C_4_–170.8 ppm) and H_3_ and two carbons (C_4_–170.8 ppm; C_6_–176.4 ppm), as well as one correlation point between H_1_ and one carbon (2:1:2). Full spectra available in Supplemental Figure S10.

To gain further insight into the coordinating moieties of the HG_3_ ligand, 2D heteronuclear single quantum coherence (HSQC) and heteronuclear multiple bond correlation (HMBC) were utilized; HSQC and HMBC of the free HG_3_ ligand are shown in Figure 4.

**Figure 5:**
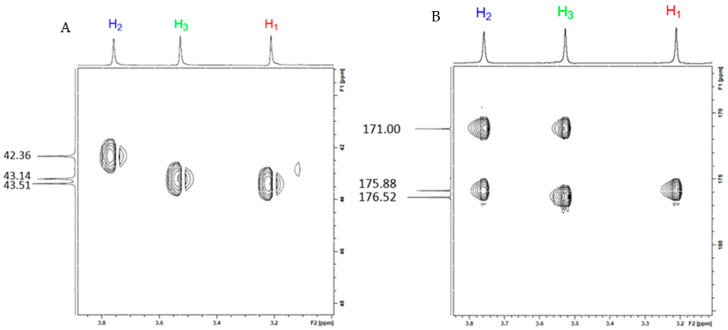
2D HSQC of MgG_3_ (**A**) showing the same observable nuclei points that were observed for the free HG_3_ ligand and the HMBC of MgG_3_ (**B**) indicating a change in the observable nuclei pattern; the observable nuclei now show a 2:2:1 pattern, which indicates that the H_1_ proton is now the most upfield shifted proton. Full spectra available in Supplemental Figure S11.

Subsequently, 2D HSQC and HMBC were conducted on MgG_3_. The most significant change in the spectrum was observed for the H_3_ proton (Figure 5), which supports that the terminal amine is also involved in magnesium chelation. The combination of both the 1D- and 2D-^1^H-NMR studies suggests that all available Lewis bases of HG_3_ are coordinated to the magnesium metal via an N_3_O donor set, as the formation of a bidentate ring structure of this size is unlikely and implicates HG_3_ as a tetradentate chelate with the Mg^2+^ cation. This is further supported by the presence of a protonated backbone amide stretch [50] observed on the spectra of the HG_3_ ligand FT-IR that is not observed for the MgG_3_ complex (Figure S2).

#### 2.1.3. Determination of the Chemical Composition of MgG_3_ via TGA/DSC and EA

Magnesium characteristically exhibits an extensive hydration sphere [1,51] and assumes a six-coordinate, octahedral geometry—especially in the presence of biologically relevant ligands [52,53,54]. It was hypothesized then, that the remaining two coordination sites of the magnesium remaining beyond the HG_3_ ligand donor set (N_3_O) would be occupied by water, while charge balance would be achieved via a hydroxy anion. To confirm this model, solid state characterization via TGA, DSC, and EA were employed.

**Figure 6:**
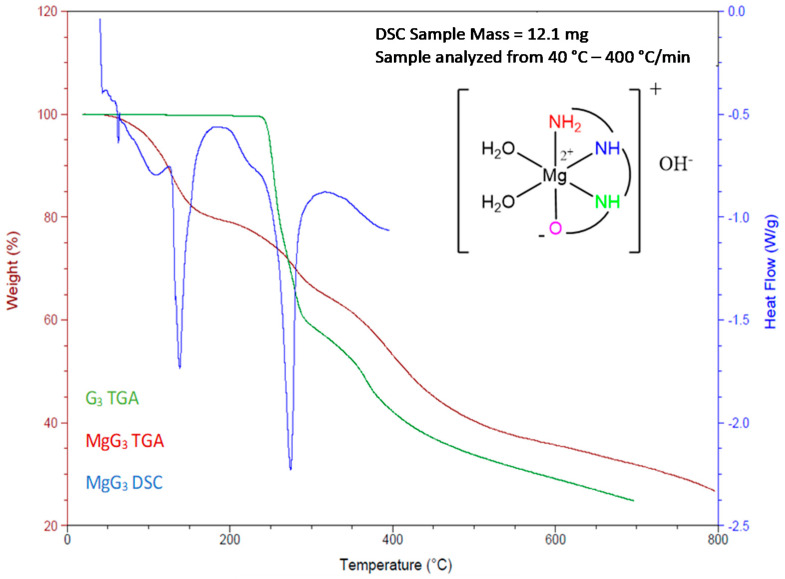
Solid state characterization of MgG_3_ showing the TGA of HG_3_ (**green**) showing only one continuous weight percent change beginning at 240 °C, the TGA of MgG_3_ (**maroon**) showing multiple weight percent changes, further highlighted by the three observable endotherms of the DSC of MgG_3_ (**blue**).

The TGA of MgG_3_ was strikingly different than that of the free HG_3_ ligand, which exhibited only one continuous weight percent change beginning at approximately 240 °C. MgG_3_ exhibited three weight percent changes: 6.6% at 109 °C, 14.1% at 191.5 °C, and one continuous change at approximately 240 °C (Figure S3). The first weight percent change corresponds to loss of a hydroxy anion—further validated by conductivity which showed a monoanionic character of MgG_3_. The second weight percent change corresponds to the loss of two coordinating waters from a parent complex of [Mg(G_3_)(H_2_O)_2_]OH, and the last corresponds to the degradation of the free HG_3_ ligand. These three independent events were further supported by the presence of three separate observable endotherms on the DSC at ~109, 191 and 240 °C (Figure 6).

The chemical composition of MgG_3_ was further verified via elemental analysis; replicate analyses were conducted to confirm percent composition. Analysis confirmed a complex of composition [Mg(G_3_)(H_2_O)_2_] with experimental values as follows: C = 29.22%, H = 6.22%, N = 16.03%, which coincide with theoretical values (Figure S5).

#### 2.1.4. Evaluating the Solubility of MgG_3_

Given the important role that solubility plays in magnesium uptake, the potential solubility of MgG_3_ was a driving interest in exploring HG_3_ as a magnesium chelate ligand. Over triplicate independent runs, the solubility of MgG_3_ was found to be 169 ± 12.5 g/100 mL H_2_O (see Table 1). This solubility of MgG_3_ is approximately 3× greater than that of magnesium chloride, 5× more soluble than magnesium sulfate, and 8× more soluble than magnesium citrate—more commonly used magnesium supplementation formulations. Solutions of MgG_3_ turn a yellow color upon solution saturation. Given previous studies, this increased solubility is believed to be to contribute significantly to the bioavailability of the MgG_3_ complex. Complex uptake was further evaluated via in vitro analysis in CaCo-2 cells.

### 2.2. Cellular Uptake of MgG_3_

**Figure 7:**
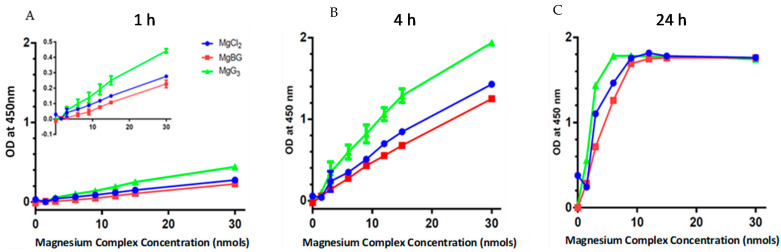
Cellular uptake of MgG_3_ (**green: 9% Mg**), MgBG (**red: 14% Mg**), and MgCl_2_ (**blue: 25% Mg**) in CaCo-2 cells analyzed at 1 h (**A**): with included expanded inset), 4 h (**B**), and 24 h (**C**). Both 1 and 4 h time points show the increased cellular uptake of MgG_3_ relative to MgBG and MgCl_2_, with 24 h showing cell saturation.

Cellular uptake of MgG_3_ was evaluated in CaCo-2 cells relative to MgCl_2_ and MgBG, utilizing a BioVision colorimetric magnesium uptake assay kit. Cellular uptake data was collected at incubation times of 1 h (required kit incubation time), 4 h (the amount of time required for uptake in the GI), and 24 h (the amount of time required for a complex to clear the GI). It was hypothesized that the exceptional solubility of MgG_3_ (determined to be 169 ± 12.5 g/100 mL over triplicate independent runs), that stems from the coordination of the triamino acid HG_3_, would result in increased bioavailability and a subsequent increase in cellular uptake. Cellular uptake data indicate that MgG_3_ exhibits greater cellular uptake than MgBG and MgCl_2_ at a significantly lower percent composition of magnesium (9, 14 and 25%, respectively) and that uptake maintains linearity (Figures S16 and S18)—uptake relative to %Mg composition is shown in Supplemental Data (Figure S17). This greater level of uptake was observed at both 1 and 4 h. At 24 h, data indicated that cell saturation had occurred (Figure 7). 

Kinetic evaluation of cellular uptake was also conducted to determine how uptake of MgG_3_ compared to that of MgBG and MgCl_2_. Kinetics were evaluated utilizing the slope of the line of uptake of the complex in the CaCo-2 cells. Kinetics of uptake were evaluated at 1 and 4 h—kinetics evaluation at 24 h was not pertinent, due to the observable saturation of the cells indicated by the plateau of the uptake curve at magnesium concentrations greater than 10 nmols. At 1 h, MgG_3_ exhibited uptake approximately 1.6× faster than MgCl_2_, and approximately 1.9× faster than that of MgBG. At 4 h, MgG_3_ showed uptake that was approximately 1.4× faster than that of MgCl_2_, and approximately 1.5× faster than that of MgBG (Figure S16). It should also be noted that at 4 h, MgG_3_ uptake had plateaued (at 30 nmols), while both MgCl_2_ and MgBG had not. This suggests that MgG_3_ achieves cellular uptake faster than both MgCl_2_ and MgBG.

## 3. Discussion

The focus of the work was to produce a fully characterizable, highly soluble magnesium complex that could provide greater bioavailability than current magnesium nutrient compounds and be fully characterizable in the solution and solid state to allow for both nutraceutical and pharmaceutical development.

**Scheme 1:**
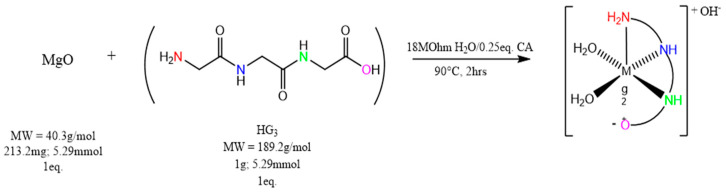
Synthesis of MgG_3_ Synthesis was conducted at 1:1 molar equivalent of MgO and HG_3_, a 0.25 eq of citric acid (CA) was added to aid in MgO solubility.

Synthesis of MgG_3_ was carried out in the presence of citric acid in hopes that it would aid in the solubility of the magnesium oxide starting material by providing a proton source as well allowing the formation of the significantly more soluble magnesium citrate. As was observed, this is the case given the lack of solubility observed for magnesium oxide in solution, and the subsequent increase in observed solubility for the combined solution of magnesium oxide, citric acid, and triglycine. It is believed that after the combination of magnesium oxide, citric acid, and triglycine, that there is a kinetic equilibrium achieved that results in the formation of magnesium triglycine and a trace amount of magnesium citrate, that results in the increased solubility. Syntheses conducted in the citrate buffer (0.25 M), indicated that magnesium citrate predominates, as was determined by ^1^H-NMR (Figure S8) and FT-IR (Figure S12). Presence of magnesium citrate indicated that synthesis must be conducted at lower citric acid equivalents—with a quarter equivalent of citric acid determined to be ideal (see Scheme 1). The preliminary synthesis of MgG_3_ was tracked via ^1^H-NMR. After two hours, there was no presence of the original three HG_3_ proton singlets, and no further shifting of the MgG_3_ proton singlets was observed. These observations indicated that reaction completion was achieved by 2 h. The near stoichiometric yield of the reaction is attributed to the entropic stability of the product—the HG_3_ ligand.

In view of prior literature on magnesium amino acid chelates, specifically the work of Martell et al., [45] which showed only a single proton displacement of triglycine when titrated into a solution of Mg^2+^ in the form of magnesium chloride, it was hypothesized that the HG_3_ ligand would coordinate predominantly through the deprotonated carboxylic acid moiety, which was initially supported using FT-IR (Figure S2)—which coincides with reported literature IR values [55], although alternate coordinating modes were possible given the presence of multiple Lewis bases in the HG_3_ ligand. Further support for this claim comes from similar observed speciation of triglycine in tin complexes as reported by Jabbari et al. [56] that showed a deprotonation of the carboxylic acid moiety at alkaline pH (≥8). ^1^H and ^13^C-NMR, both 1D and 2D, were employed to determine what Lewis base moieties were participating in coordination with magnesium. If the magnesium coordinated solely through the carboxylic acid, then only the proton adjacent to the carboxylic acid would show a significantly observable upfield shift. As the data shows, all three proton singlets exhibit an observable upfield shift (Figure 3). Furthermore, ^1^H-NMR studies of HG_3_ and MgG_3_ in DMSO-d^6^ showed the absence of proton signal splitting for the proton of the carboxylic acid for the MgG_3,_ indicating that the carboxylic acid has become deprotonated and coordinated to the magnesium metal. Shifting of all proton signals, including the significant upfield shift of the proton adjacent to the terminal amine, and the implication of the carboxylic acid coordination suggests that all available Lewis base moieties have coordinated, thus indicating that the HG_3_ ligand acts as a tetradentate ligand, which is likely given the observed torsion angles of the free HG_3_ ligand, that vary by 5–10° when protonated or deprotonated, as reported by Ivanova et al. [55], and as such would be favored entropically. Observed shifting of all proton signals is also consistent with tetradentate ligand coordination as any shift, either upfield or downfield, this is not solely indicative of all Lewis bases participating in magnesium chelation as coordination of magnesium to acids has been shown to exhibit an effect on not only the α proton, but on β and γ protons as well; with diminishing impact the farther the proton is from the coordination site [47,48,49]. This conclusion coincides with findings first reported by Martell et al. [57] and later supported by Van der Helm et al. [58] Martell showed, via titration experiments, that HG_3_ acted as a tetradentate ligand with copper and cobalt [57]. Martell’s findings also supported two bound water molecules when in aqueous solution [57]. Van der Helm showed that HG_3_ also acted as a tetradentate chelate in the presence of calcium via crystal diffraction [58]. Both researchers concluded that the HG_3_ ligand participated in coordination via an N_3_O donor set [57,58].

Chemical composition determination of MgG_3_ was carried out utilizing TGA/DSC and elemental analysis. The TGA of both HG_3_ and MgG_3_ showed stability until about 240 °C—most likely due to the extensive hydrogen bond network of the ligand as described by Srikrishnan et al. [59].

While there are many physiological factors that have been shown to contribute to magnesium bioavailability during supplementation, such as age [39], dose load [60], and frequency [61,62], there are also chemical factors that impact magnesium bioavailability, such as chemical composition and solubility [34,35,36,63]. The solubility of MgG_3_ was found to be approximately 169 ± 12.5 g/100 mL H_2_O, which is approximately three times more soluble than the next closest magnesium supplement (MgCl_2_ at 54 g/100 mL—see Table 1) and, as such, shows great promise for cellular uptake, as it is believed this increase in solubility will aid in bioavailability. As was hypothesized, the solubility of MgG_3_ was integral in generating significant cellular uptake—with MgG_3_ showing greater cellular uptake than both MgCl_2_ and MgBG at a lesser overall magnesium percent composition. Additionally, this greater observed uptake may be attributed to complex stability imparted predominantly by the tetradentate HG_3_ ligand. This increased stability is observed in the significantly decreased percent weight change slope of the MgG_3_ TGA relative to that of the steeper slope observed for the TGA of the free HG_3_ ligand, which shows a more rapid decomposition. Further support for MgG_3_ complex stability comes via ^1^H-NMR analysis in 6:1 H_2_O:D_2_O at room temperature. Over a 72 h period, the ^1^H-NMR of MgG_3_ showed little to no change, suggesting that the complex is maintained for at least this amount of time at room temperature (Figure S19).

With the synthetic ease, solubility, and cellular uptake of the MgG_3_ complex, it is hypothesized that complexation of the HG_3_ ligand to other biorelevant, metal ions (e.g., calcium, zinc) may exhibit similar properties. It is further proposed that other tripeptide sequences utilizing an N_3_O donor set may also provide entropic stability and charge balance, thus forgoing the requirement to have a counterion (as is the case of the hydroxy anion described within this work). Removal of ionic character from the overall complex may, in turn, have a profound effect on complex solubility and bioavailability, thus significantly altering cellular uptake. Future work aims at the successful synthesis and characterization of both zinc- and calcium-G_3_ complexes, and the evaluation of cellular uptake utilizing a CaCo-2 cell line. Subsequently, use of the dianionic tripeptide, Asp-Gly-Asp, as a chelate ligand for magnesium, zinc, and calcium will be conducted, and subsequent cellular uptake evaluated.

## 4. Materials and Methods

### 4.1. Materials

Magnesium oxide (99.99% metals basis) was purchased through Fisher Scientific (Hampton, NH, USA). Triglycine (Gly-Gly-Gly, BioUltra^TM^, ≥99.0%) and citric acid (ACS Reagent, ≥99.5%) were purchased through Sigma-Aldrich (St. Louis, MO, USA). 18.2 MΩ Ultrapure spectroscopic grade water was obtained in-house. D_2_O and DMSO-d_6_ NMR solvents and 200 proof anhydrous ethanol were purchased from Sigma-Aldrich (St. Louis, MO, USA). Potassium bromide (KBr) for FT-IR analysis was purchased from Sigma-Aldrich (St. Louis, MO, USA). Magnesium uptake colorimetric assay kits (includes magnesium enzyme mix, assay developer, and magnesium assay buffer) were purchased from BioVision (Milpitas, CA, USA; Catalog #385-100). Stock solutions for magnesium uptake assays were made in-house, with MgCl_2_ (BioReagent, ≥97.0%) purchased from Sigma-Aldrich (St. Louis, MO, USA) and magnesium *bis*glycinate (MgBG) provided by Balchem Corp (New Hampton, NY) and confirmed for purity in-house by ^1^H-NMR (see Supplemental Figure S1). HEK-293 (ATCC^®^ CRL-1573^TM^) cells, CaCo-2 (ATCC^®^ HTB-37^TM^) cells, and Dulbecco’s Modified Eagle Medium (ATCC^®^ 30-2002) were purchased from ATCC (Manassus, VA, USA). Penicillin streptomycin (10,000 U/mL), fetal bovine serum (FBS), and trypsin/EDTA (0.25%) were purchased from Gibco™ (Waltham, MA, USA). Clear 96-well Armadillo PCR assay plates (Catalog #AB2396) were purchased from ThermoFisher (Waltham, MA, USA).

### 4.2. Methods

#### 4.2.1. Characterization of MgG_3_

Electrospray ionization mass spectrometry was carried out on a Shimadzu 8040 LC-MS/MS—samples were analyzed utilizing a solvent system of 50% H_2_O/50% MeOH/0.1% TFA at a flow rate of 0.2 mLs/min over a 1.5 min time frame and evaluated from 0 to 600 *m/z*. 1D- and 2D-NMR were conducted on a Bruker Avance III HD 400 MHz instrument. FT-IR was carried out on a Nicolet Infrared Spectrophotometer. TGA was carried out on a TA Instrument Q500 from 20 to 800 °C with sample weights between 5 and 10 mg (weight percent changes calculated to 6.4 and 14.5%, respectively). DSC was carried out on a TA Instrument Q2000 from 30 to 400 °C with sample weights between 10 and 20 mg. Elemental analysis was conducted by Intertek Pharmaceutical Services (Whitehouse, NJ, USA). Theoretical elemental analysis values are as follows: C = 29.00%, H = 5.68%, N = 16.90%; experimental values are: C = 29.22%, H = 6.22%, N = 16.03% (Supplemental Figure S5). Uptake of MgG_3_ in CaCo-2 was determined on a Molecular Devices FlexStation 3 (Molecular Devices). Cellular uptake data was plotted using the Graphpad Prism 8 software.

#### 4.2.2. Culturing of CaCo-2 and HEK293 Cells

CaCo-2 cells were cultured from liquid N_2_ frozen stocks and rapidly thawed to RT using a water bath at 37 °C; cryopreservation media was removed with a micropipette after cells were pelleted via centrifugation for 5–10 min at 125 g. Cells were resuspended in 1 mL of room temperature Dulbecco’s Modified Eagle Medium (DMEM) and cultured in 14 mL DMEM (total volume of 15 mL) with a seeding densities of 3.6 × 10^4^ cells/cm^2^ (CaCo-2) and 6 × 10^3^ cells/cm^2^ (HEK 293) in a T-75 cm^2^ culture flask and left to grow in an incubator at 37 °C and 5% CO_2_. Cells were sub-cultured at 90% confluency, and sub-culturing occurred a minimum of five times before use in uptake assays. When cultures reached 90% confluency, media was removed and 3 mL of trypsin/EDTA was added—the trypsinized culture flask was placed back in the incubator for ~10 min to detach cells. Once detached cells were confirmed under a microscope, the culture flask was rinsed with 6 mL of fresh media to neutralize the trypsin/EDTA—cells for uptake assays were then counted utilizing a DeNovix CellDrop™. Once counted, cells were pelleted down via centrifugation at 125 g for 5–10 min, and the neutralized trypsin-media supernatant was removed. Cells were resuspended in 5 mL magnesium assay buffer for cellular uptake assay.

### 4.3. Synthesis of Magnesium Triglycine ([Mg(G_3_)(H_2_O)_2_]OH—(MgG_3_)

A 1.0025 g sample of triglycine (HG_3_—5.29 mmol; 1 eq.) was dissolved in 10 mL of 18.2 MΩ H_2_O in a 50 mL round-bottom flask, with constant stirring at 90 °C. A separate solution of 215.5 mg magnesium oxide (MgO—5.29 mmol; 1 eq.) was taken up in 10 mL of 18 MΩ H_2_O, with an addition of 253.6 mgs of citric acid (CA—0.25 eq), constantly stirred and heated to 90 °C. The MgO/CA solution was subsequently added to the triglycine solution. Immediately upon addition, the combined solution turned a milky white color, whereupon this became a clear solution after ~20 min with stirring at 90 °C. The reaction was run for 2 h, and then cooled to room temperature. The pH of the solution was noted as 10.2. The solution was concentrated to approximately 3 mL, via rotary evaporation, and a white solid was precipitated with anhydrous ethanol. The solid was isolated by centrifugation at 4000 rpm at room temperature for 10 min, and the ethanol decanted off. The solid was triturated with copious diethyl ether to remove excess ethanol, recentrifuged as before, the ether decanted off, and the solid dried in vacuo overnight. The dried white material was collected, and the yield obtained. The solubility of MgG_3_ was determined to be 169 ± 12.5 g/100 mL H_2_O and the purity was found to be 90+% via ^1^H-NMR integration.

### 4.4. Determining Magnesium Uptake in CaCo-2 Human Cells

Sample solutions for use with the colorimetric assay were prepared in house—each sample being 150 μM. The samples tested included magnesium chloride (MgCl_2_), magnesium bisglycinate (MgBG) and MgG_3_. Each well contained a total volume of 150 μL (50 μL of cells in ultrapure H_2_O, 50 μL of buffer/enzyme/developer mix, and varying amounts of sample from 0 to 20 μL brought to total volume with 18.2 MΩ H_2_O to desired well concentration). To conduct the assay, 50 μL of cells in ultrapure H_2_O was added to each well with a multichannel micropipette. Subsequently, the required volume of sample was added to each well and brought to volume with ultrapure H_2_O. To ensure a consistent starting time point, 50 μL of the magnesium buffer/enzyme developer mix was added to each well with a multichannel micropipette. The plate was incubated for approximately 1 h and then placed in a Flexstation 3 plate-reading spectrofluorometer to analyze well absorbance at 450 nm. Each well was analyzed at 37 °C for endpoint value over nine full plate scans with 3 scans/well/plate scan (a total of 27 scans per well) and the reported value of each well was the average value of these scans after background subtraction. Data was collected at 1, 4 and 24 h. Raw data was reduced and plotted as absorbance value against the magnesium concentration of each well. All assays were repeated as triplicate independent runs—error bars shown in graph (Figure 7). All data shown as ± SEM (MgCl_2_ = 0.029, MgBG = 0.025, MgG_3_ = 0.053; Upper 95% C.I.: MgCl_2_ = 0.168, MgBG = 0.123, MgG_3_ = 0.278; Lower 95% C.I.: MgCl_2_ = 0.031, MgBG = 0.001, MgG_3_ = 0.024).

## 5. Conclusions

The facile synthesis of a new magnesium chelate, MgG_3_, was successfully accomplished at near stoichiometric yield, utilizing a magnesium oxide starting material and citric acid at a 0.5 M equivalent. The characterization of the complex via both 1D and 2D NMR showed the free ligand HG_3_ to act as a tetradentate ligand, and the overall complex to exhibit six-coordinate pseudo-octahedral geometry with the remaining two coordination sites being occupied by water, and inherent charge balance being achieved via a hydroxy anion—as was determined via TGA/DSC and elemental analysis. The complex exhibited exceptional water solubility (169 ± 12.5 g/100 mL). It is believed that this increase in water solubility, due to the triglycine ligand, promotes the observed improved cellular uptake in HEK293 and CaCo-2 cell lines, relative to currently on-the-market magnesium supplements—with MgG_3_ showing significantly greater cellular uptake in CaCo-2 cells at concentrations ranging from 0–30 mmol, with a magnesium composition of 9%, relative to 14% (MgBG) and 25% (MgCl_2_), respectively. These findings suggest MgG_3_ may be a new nutraceutical/pharmaceutical and provide positive insight into further use of naturally occurring tripeptides as metal chelates to aid in complex stability and the bioavailability of other micronutrient nutraceuticals.

## 6. Patents

U.S. Patent # 63032955 filed 6/1/2020.

## Figures and Tables

**Table 1:** The aqueous solubilities (g/100 mL) at room temperature, acid/base chemistry, molecular weight, and percent magnesium composition of common magnesium supplements.

Form (Oxide/Salt/Chelate)	Acid/Base Chemistry	Solubility (g/100 mLs H_2_O)	MW g/mol (%Mg)	Reference
Magnesium Oxide	Alkaline	0.010	40.30 (60.3)	[25]
Magnesium Citrate	Acidic (pKa_1_ = 3.13)	20	214.41 (11.3)	[26]
Magnesium Chloride	Neutral	54.0	95.21 (25.5)	[27]
Magnesium Sulfate	Acidic (pKa_1_ = 3.0; pKa_2_ = 1.99)	35.7	120.37 (20.1)	[25]
Magnesium Orotate	Acidic (pKa_1_ = 2.83)	Slightly Soluble	334.48 (7.2)	[28]
Magnesium Taurate	Acidic (pKa = 1.50)	Slightly Soluble	272.57 (8.9)	[29]
Magnesium Aspartate	Acidic (pKa_1_ = 1.88; pKa_3_ = 3.65)	4.0	288.49 (8.5)	[30]
Magnesium Threonate	Acidic (pKa_1_ = 3.4)	Soluble	294.50 (8.3)	[31]
Magnesium Malate	Acidic (pKa_1_ = 3.46; pKa_2_ = 5.10)	Slightly Soluble	156.37 (15.5)	[32,33]
Magnesium *Bis*glycinate	Acidic (pKa = 2.34)	1.36	172.42 (14.1)	In-house
Magnesium Hydroxide	Alkaline	0.00069	58.32 (41.7)	[25]
Magnesium Carbonate	Weakly Alkaline	0.18	84.31 (28.8)	[26]
Magnesium Triglycine	Acidic	169	265.50 (9.1)	This Work

## Data Availability

The data presented in this study are available on request from the corresponding author.

## Supplementary Materials

Supplementary materials are provided as a Microsoft Word document. The document includes supplemental MgG_3_ characterization data, full spectra ^1^H/^13^C-NMR of HG_3_/MgG_3_, synthetic conditions evaluation at higher citric acid concentrations via ^1^H/^13^C-NMR, and uptake of MgG_3_ in HEK293 cells.

## Sample Availability

Samples of the compound MgG_3_ are available, within reason and pending approval from Balchem Corp., from RPD at Syracuse University.
