# Supplementary material for: Synthesis and Chemical and Biological Evaluation of a Glycine Tripeptide Chelate of Magnesium
Source: Molecules. 2021 Apr 21;26(9):2419. doi: 10.3390/molecules26092419 (PMC8122334; doi:10.3390/molecules26092419)
Supplement: Supplementary file 1 [file molecules-26-02419-s001.zip › molecules-1164852-supplementary.pdf]

Supplemental Information

Synthesis and Chemical and Biological Evaluation of a Glycine Tripeptide Chelate of Magnesium-

Derek R. Case, Jon Zubieta, Ren Gonzalez and Robert P. Doyle

Table of Contents

^{1}H NMR of Magnesium *Bis*glycinate **(S1)**……………………………………………...**Page 2**

FT-IR of G_3 and MgG_3 **(S2)**………………………………………………………......**Page 3**

DSC of MgG_3 **(S3)**...…………………………………………………………...……**Page 4**

TGA of MgG_3 **(S4)**……………………………………………………………………...**Page 5**

Elemental Analysis of MgG_3 **(S5)**…………………………………………………...**Page 6**

^{1}H NMR of HG_3 Ligand and MgG_3 Complex **(S6)**……………………………...**Page 7**

^{1}H NMR Overlay of HG_3 and MgG_3 Full Spectra **(S7)**……………………………...**Page 8**

^{1}H NMR of MgG_3 Synthesis in Citrate Buffer **(S8)**...…………………………...……**Page 9**

^{1}H NMR of HG_3 and MgG_3 in DMSO-d6 **(S9)**……………………………………....**Page 10**

Full Spectra HSQC and HMBC of HG_3 **(S10)**…………………………………………...**Page 11**

Full Spectra HSQC and HMBC of MgG_3 **(S11)**……………………………………...**Page 12**

FT-IR of Magnesium Citrate Precipitate **(S12)**………………...…………………....**Page 13**

^{13}C NMR of G_3 and MgG_3 **(S13)**……………………………………………...…**Page 14**

Uptake of MgG_3 in HEK293 Cells **(S14)**………...…………………………………....**Page 15**

Uptake of MgG_3 Relative to % Composition Magnesium in HEK293 Cells **(S15)**.....**Page 16**

Linear Regression of Cellular Uptake of MgG_3 **(S16)**……………………………....**Page 17**

Uptake of MgG_3 Relative to % Composition Magnesium in CaCo-2 Cells **(S17)**........**Page 18**

Kinetics of MgG_3 Uptake in CaCo-2 Cells **(S18)**…………………………...…..**Page 19**

^{1}H NMR Stability Study of MgG_3 Over 72 hours **(S19)**……………………………**Page 20**

^{1}H NMR of MgG_3 Synthesis Conducted with Increasing Citric Acid **(S20)**………..**Page 21**

S1

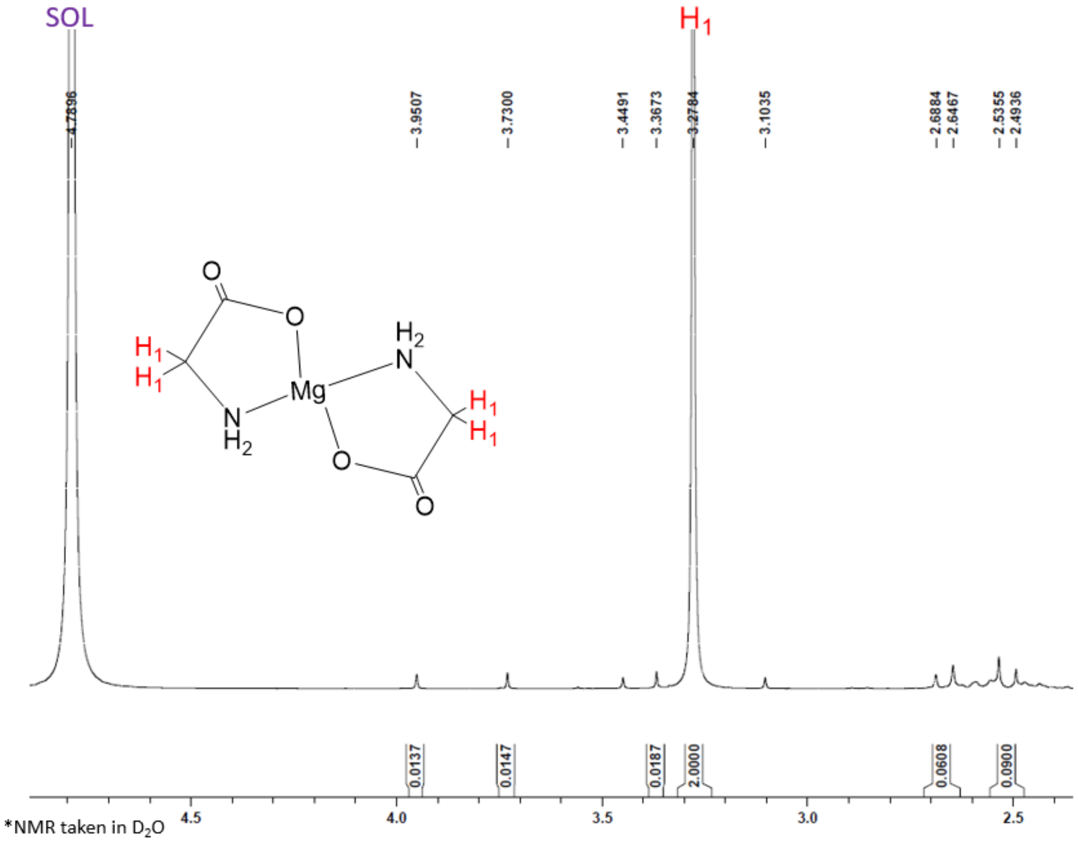

S 1. ^{1}H NMR of magnesium *bis*glycinate supplied by Balchem Corp (New Hampton, NY) of 95.5% purity, showing the presence of 4.5% magnesium citrate.

S2

Region of Interest	Functional Group	G_3	MgG_3
$950cm^{-1}$ - $1250cm^{-1}$	C-O	$991cm^{-1}$	$1028cm^{-1}$
$2500cm^{-1}$ - $2700cm^{-1}$	O-H (Carboxylic Acid)	$2635cm^{-1}$	Has been deprotonated
$2000m^{-1}$ - $2150cm^{-1}$	R-NH_2-R	$2090cm^{-1}$	N/A

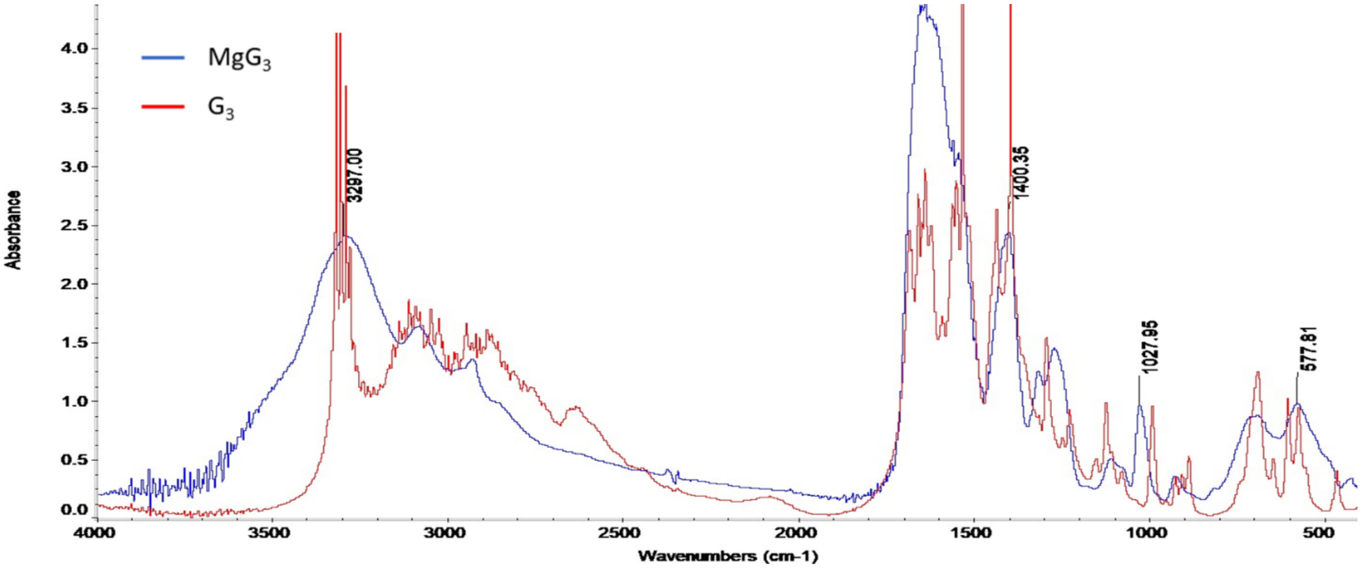

S 2. FT-IR of MgG_3 showing G_3 (red) and MgG_3 (blue). Infrared shows the presence of the -COOH proton for the free G_3 ligand, and the subsequent disappearance of this proton for complexed MgG_3 as observed by the flattening of the broad band observed for G_3 at approximately $2600cm^{-1}$. IR also shows a shift of the C-O stretch of the -COOH moiety from $991cm^{-1}$ for the free G_3 ligand to $1028cm^{-1}$ for the complex MgG_3. Additionally, there is a disappearance of the R-NH_2-R moiety stretch at approximately $2100cm^{-1}$

S3

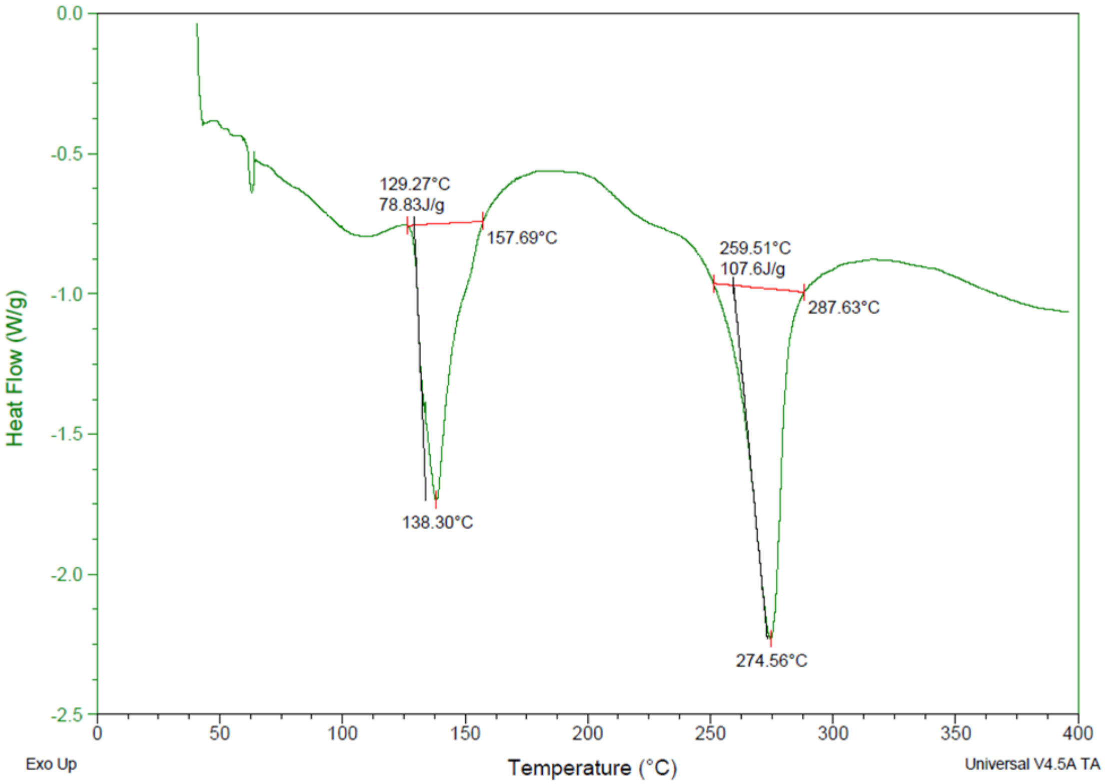

S 3. DSC of MgG_3 showing three independent endotherms that are consistent with the loss of an anion, two waters and the free G_3 ligand. Evidence supports a composition of MgG_3 containing two waters and a hydroxide anion. Mg-H_2O complex endotherms are supported by the literature.[1]

S4

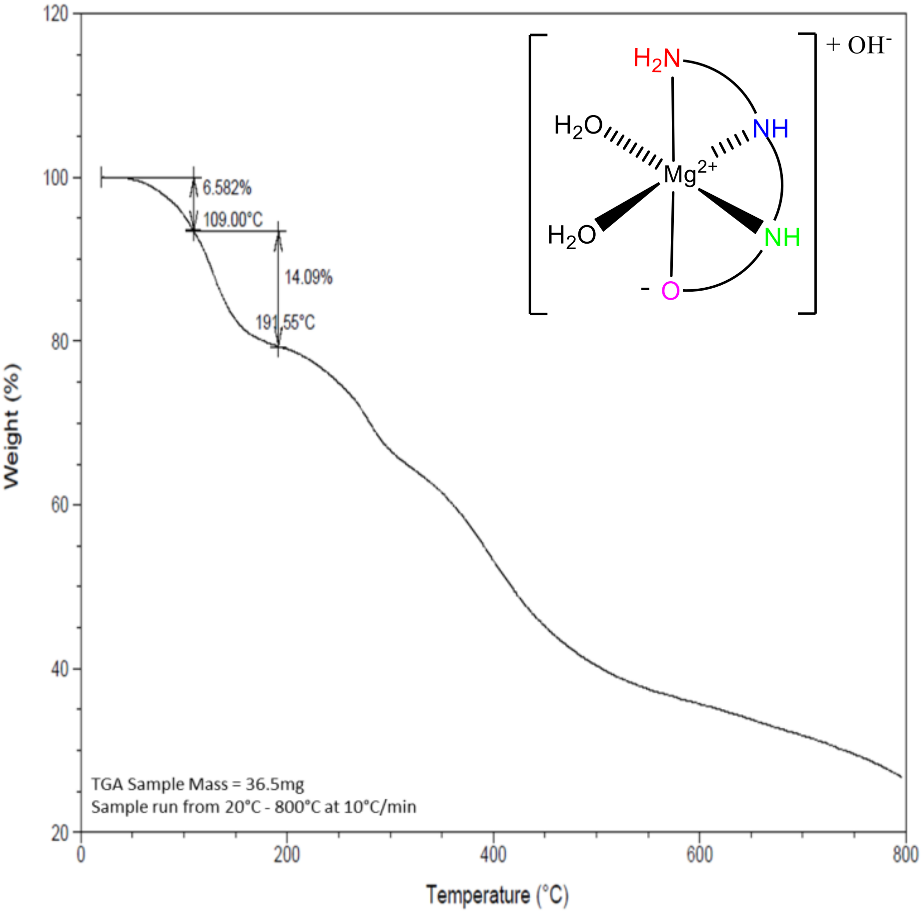

S 4. TGA of the MgG_3 complex showing two percent weight decreases attributed to the hydroxy anion and the two coordinated waters.

S5

Elemental Analysis Values			
MgG_3	Carbon %	Nitrogen %	Hydrogen %
Calculated Values	29.00%	16.90%	5.68%
Sample 1	29.25%	16.03%	6.31%
Sample 2	29.19%	16.02%	6.12%
Average Mass %	29.22%	16.03%	6.22%
Average Error	0.22%	-0.87%	0.54%

S 5. Duplicate Elemental Analysis values confirming the chemical composition of MgG_3.

S6

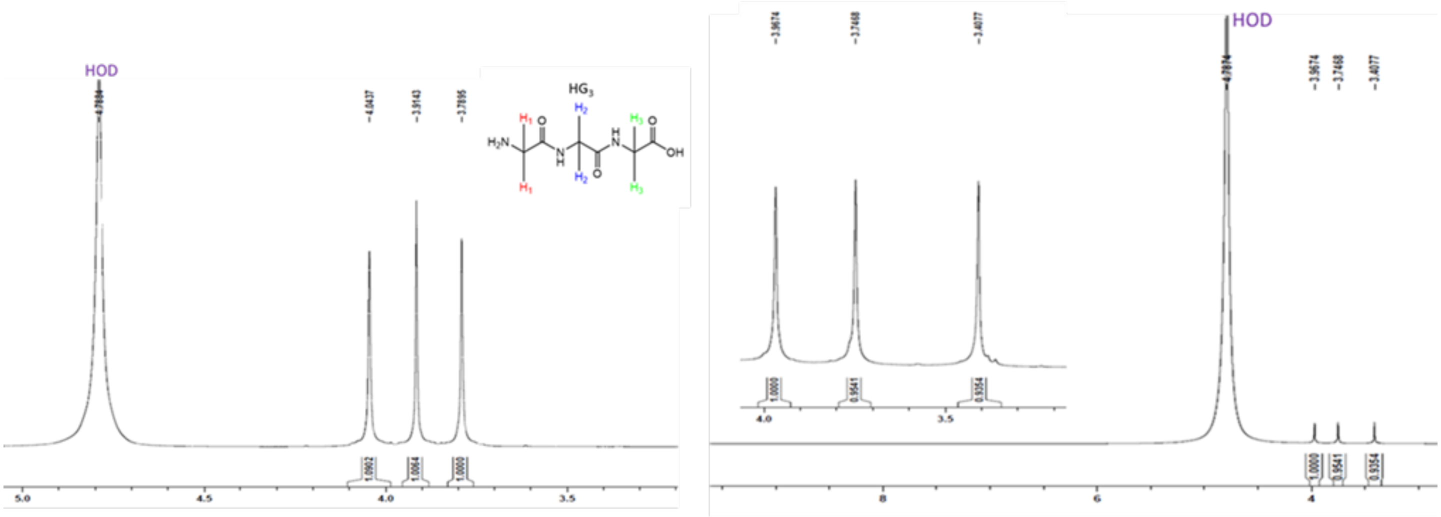

S 6. ^{1}H NMR of HG_3 (**left**) and MgG_3 (**right)** showing three pure singlets of desired integration – with upfield shifting of the MgG_3 complex signals.

S7

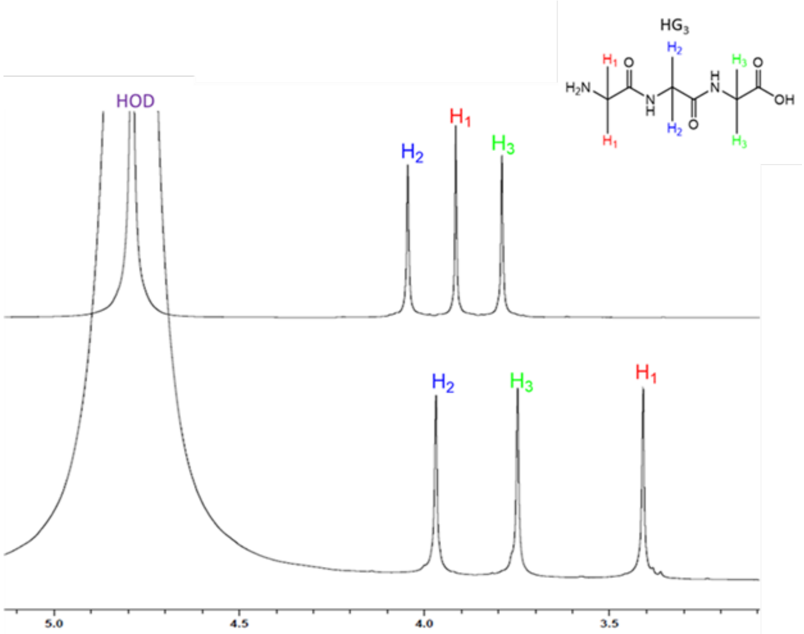

S 7. ^{1}H NMR overlay of HG_3 (**Top**) and MgG_3 (**Bottom**) including solvent peak for shift verification.

S8

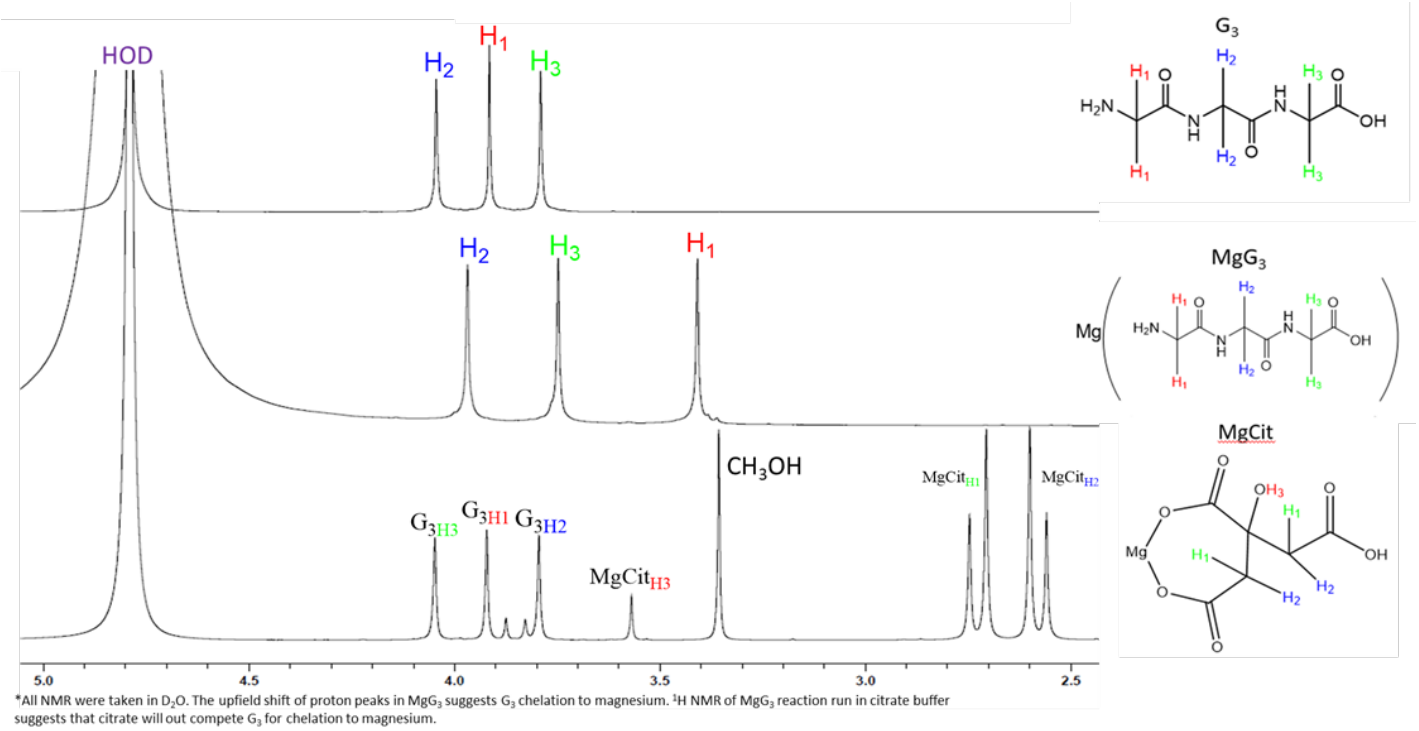

S 8. ^{1}H NMR of MgG_3 synthesis in reported stoichiometric conditions and citrate buffer showing propagation of reaction toward magnesium citrate when conducted in citric acid buffer.

S9

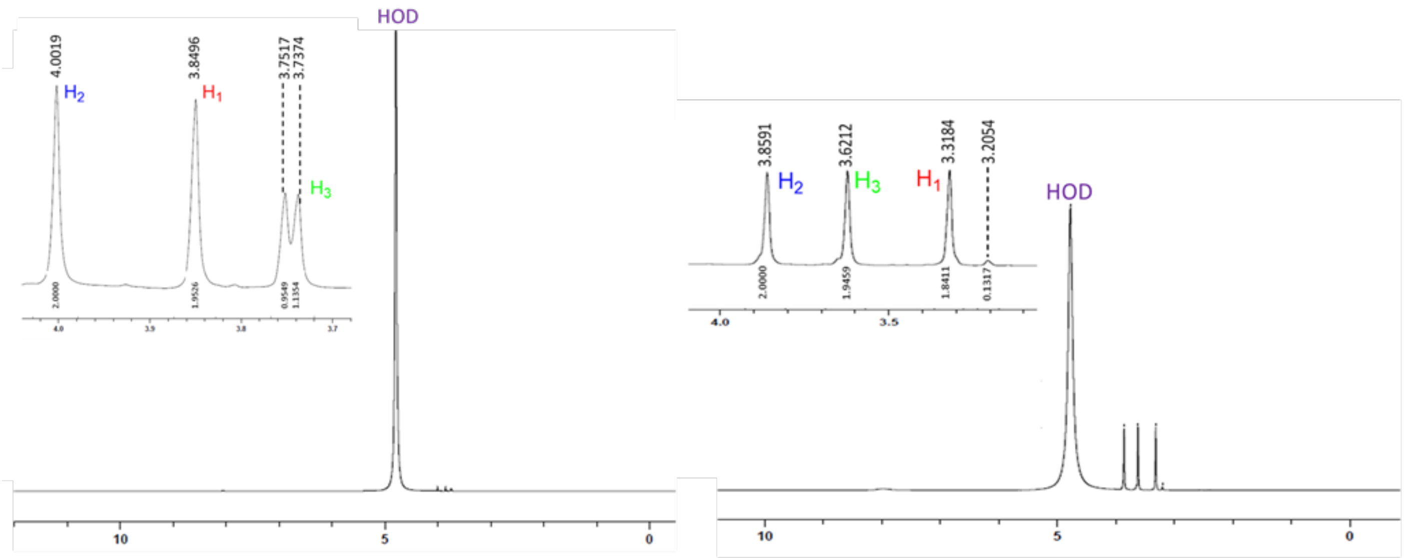

S 9. Full spectra ^{1}H NMR of HG$_3$ (**Left**) and MgG$_3$ (**Right**) in DMSO-d6

S10

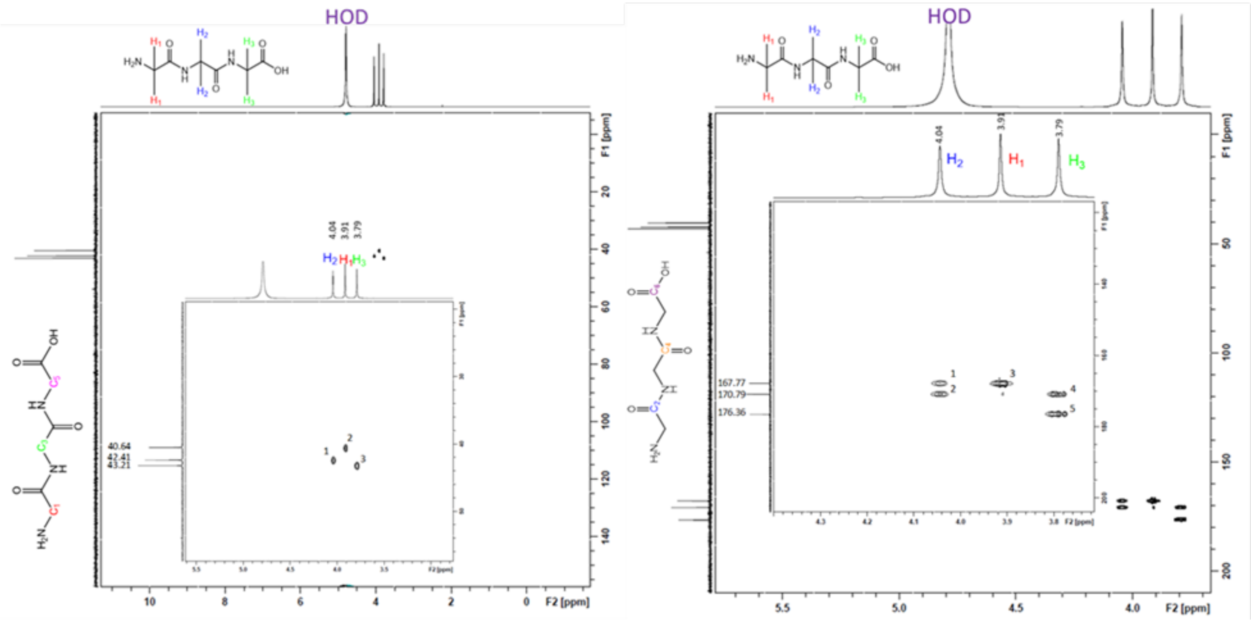

S 10. Full spectra HSQC (**Left**) and HMBC (**Right**) NMR of the free HG_3 ligand in a 6:1 H_2O:D_2O solution

S11

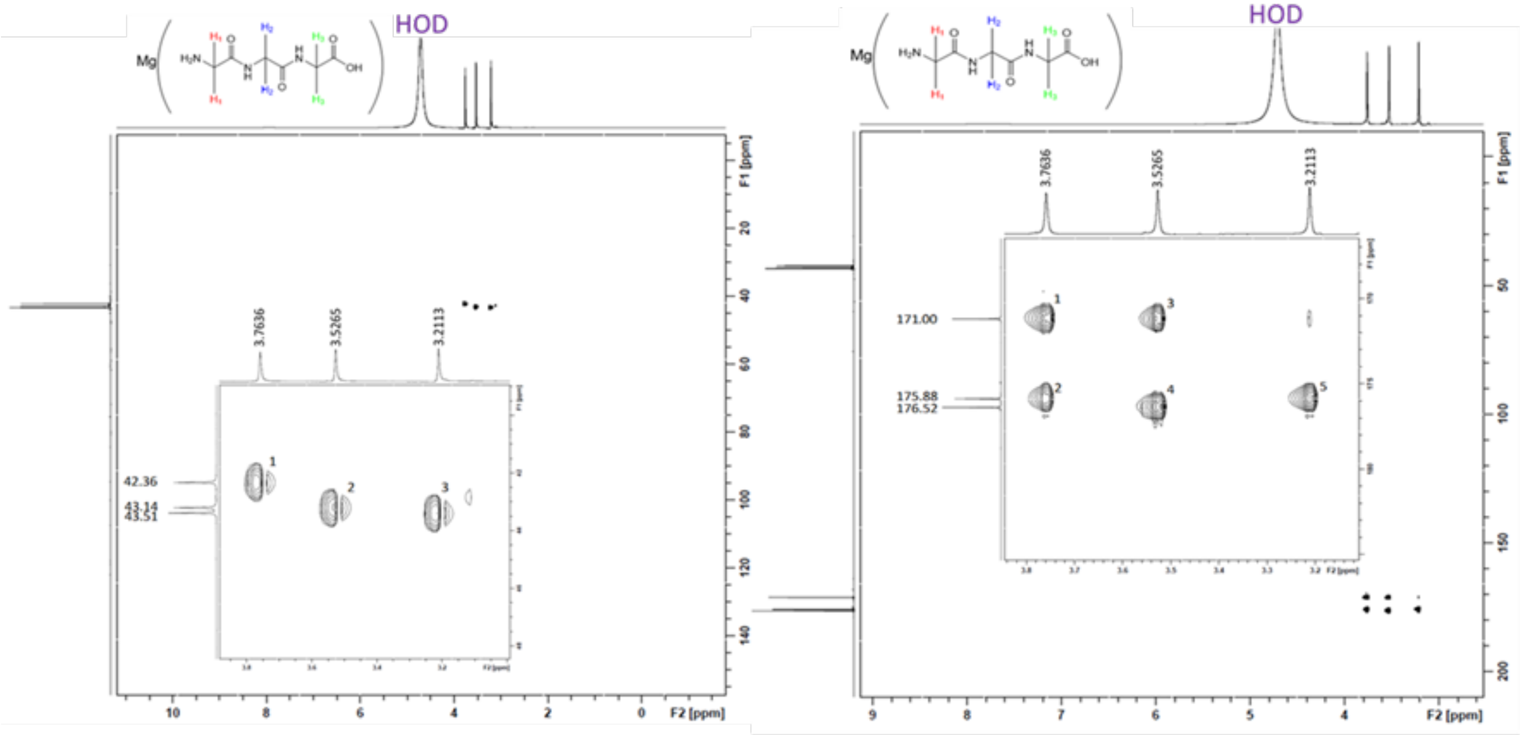

S 11. Full spectra HSQC (**Left**) and HMBC (**Right**) of MgG_3 in a 6:1 H_2O:D_2O solution

S12

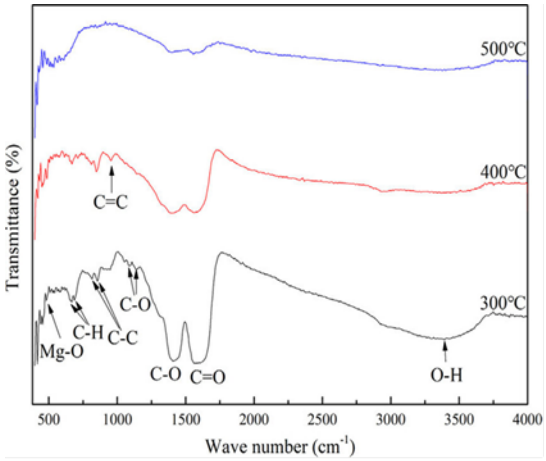

Taken from: Ding et *al.* DOI: 10.1016/j.ceramint.2018.12.098

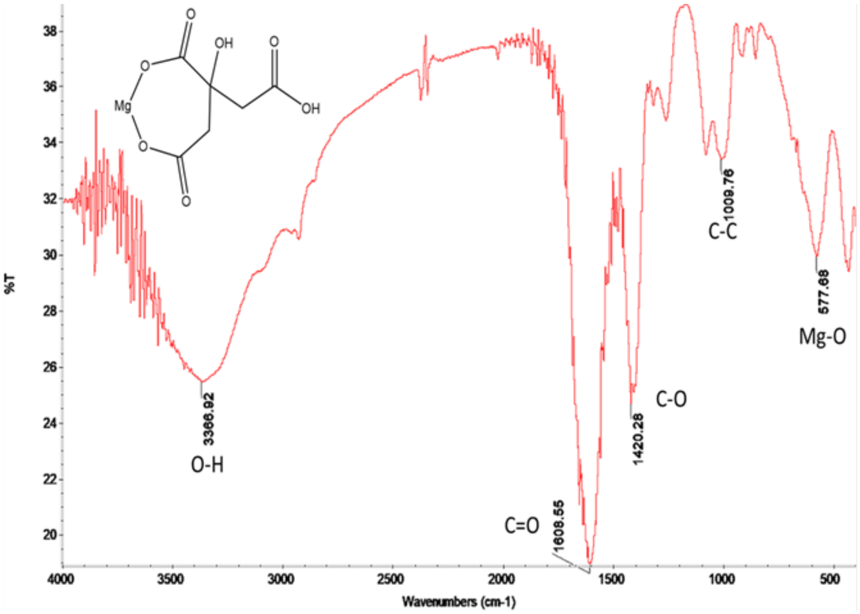

S 12. FT-IR of Precipitate Isolated from MgG_3 synthesis conducted in citrate buffer coincides with literature reports of magnesium citrate.[2]

S13

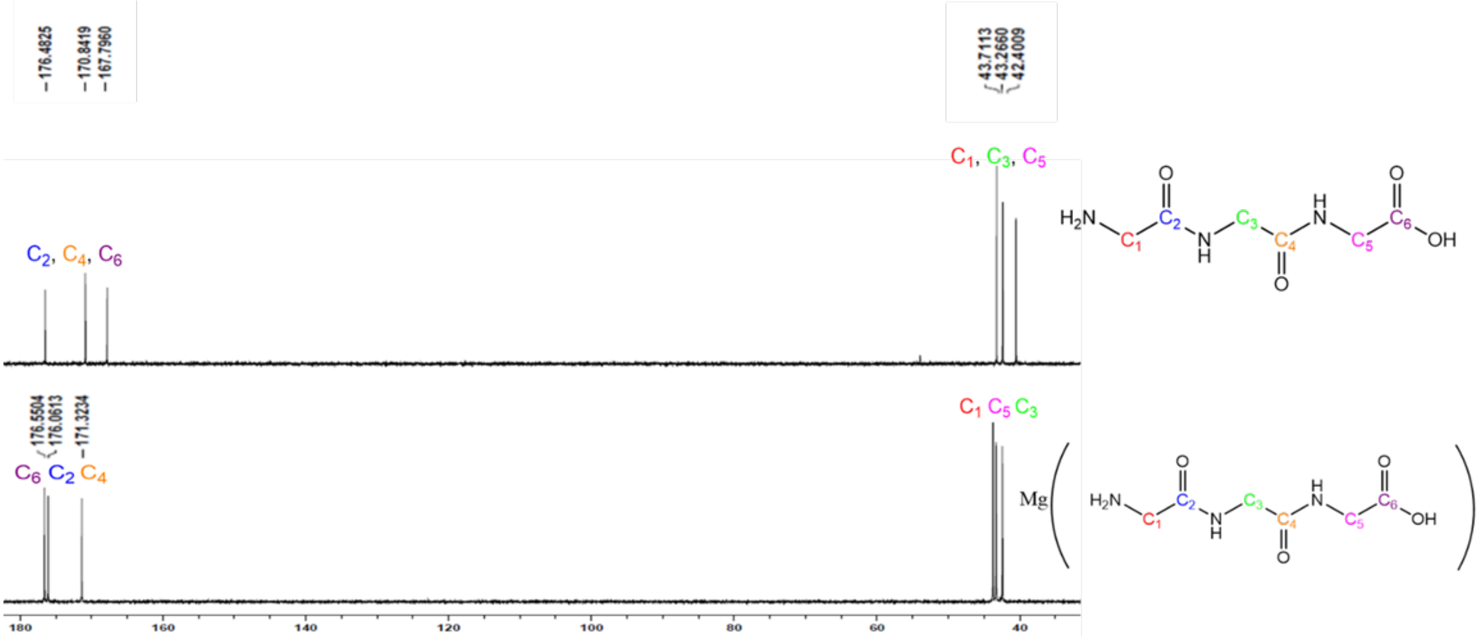

S 13. ^{13}C NMR of the free G_3 ligand (top) and the complex MgG_3 (bottom) – as expected, G_3 shows six carbons. The subsequent complexation of G_3 to magnesium also shows six carbons but shows a subsequent down-field shift of carbon signals, as is expected given the upfield shift of the proton signals.

S14

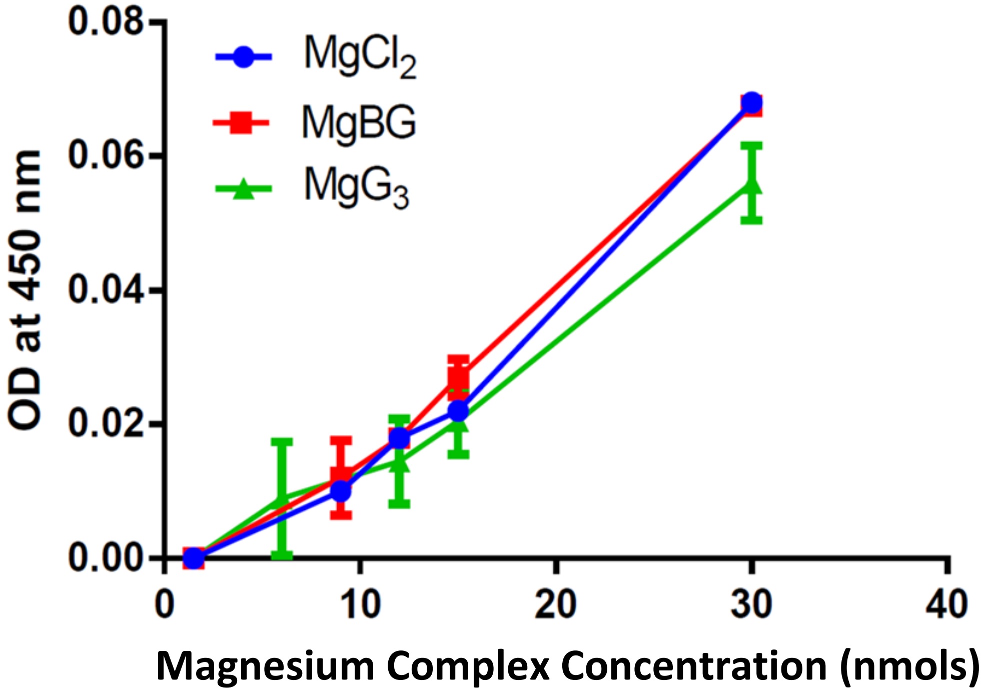

S 14. Cellular uptake of MgG_3 in HEK293 Cells with MgG_3 showing uptake comparable to that of both magnesium chloride (blue) and Balchem's magnesium bisglycinate (red).

S15

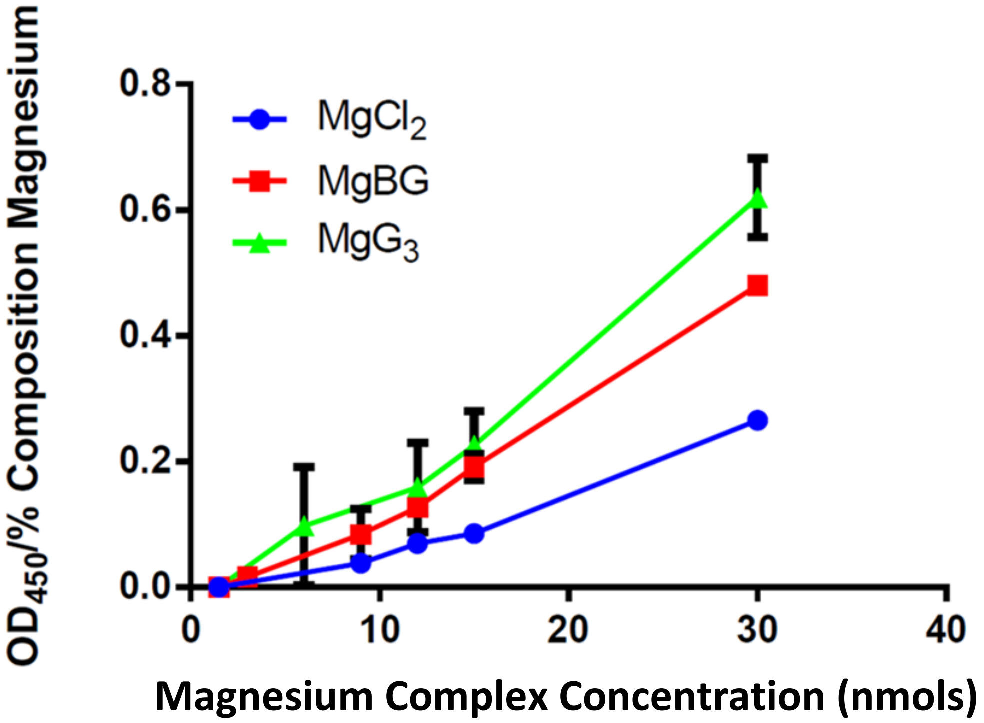

S 15. Replot of S6 showing magnesium uptake for MgG_3 (green), MgBG (red), and $MgCl_2$ (blue) in HEK293 cells against percent magnesium composition. At only 9% magnesium composition, MgG_3 shows uptake comparable to that of both MgBG and $MgCl_2$.

S16

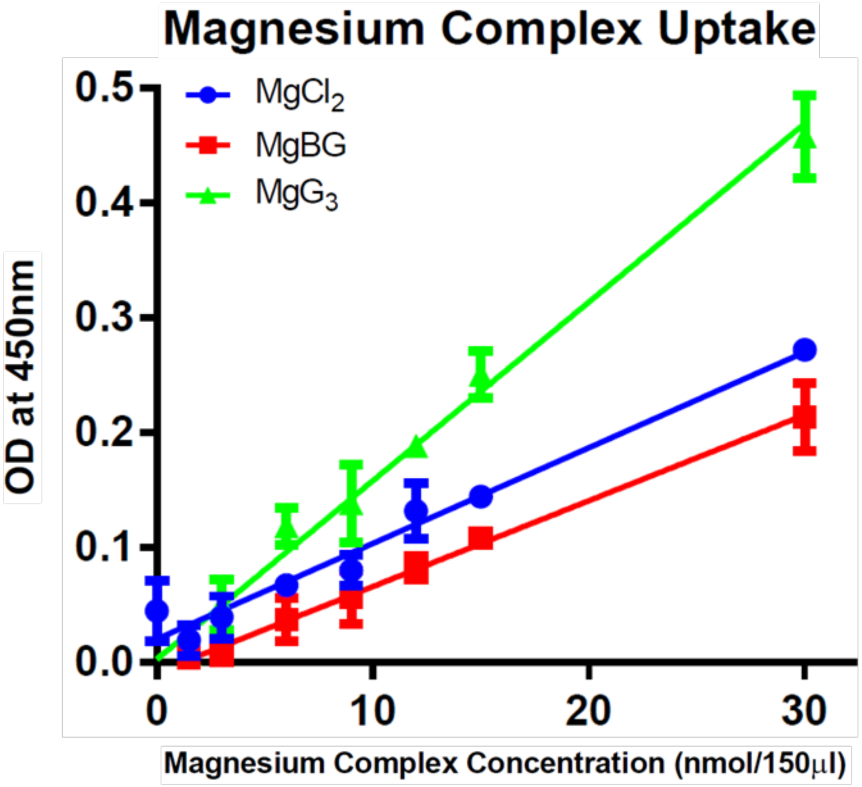

S 16. Linear regression of $MgCl_2$, MgBG, and MgG_3 in CaCo-2 cells showing that uptake maintains linearity for all magnesium compounds between 0 – 30 nmol in 150μl. R^2 values are 0.9461, 0.9635, and 0.9766 for $MgCl_2$, MgBG, and MgG_3 respectively.

S17

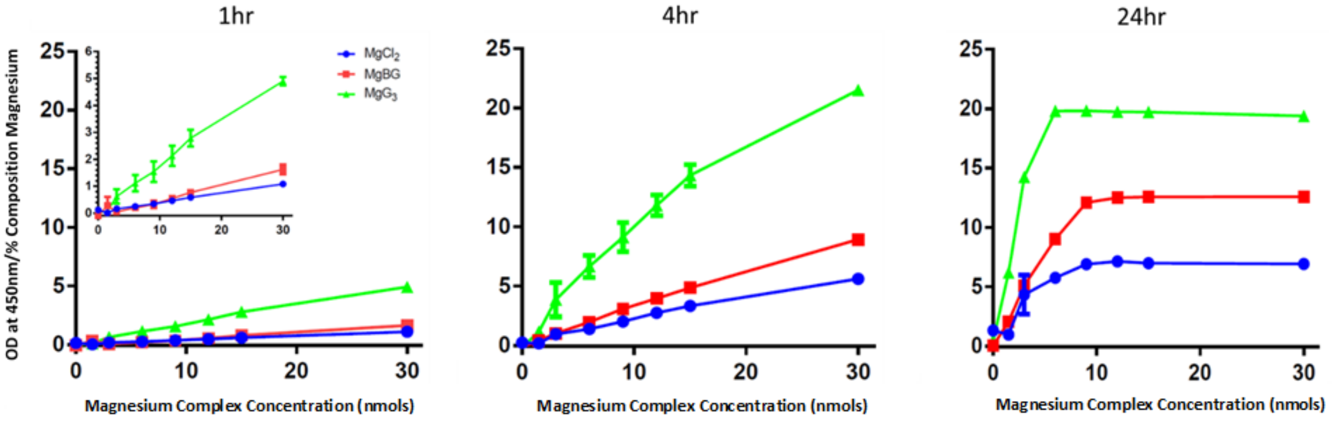

S 17. Replot of Figure 7 showing magnesium uptake for MgG_3 (green), MgBG (red), and $MgCl_2$ (blue) in CaCo-2 cells against percent magnesium composition. At only 9% magnesium composition, MgG_3 shows greater cellular uptake per percent magnesium relative to that of both MgBG and $MgCl_2$.

S18

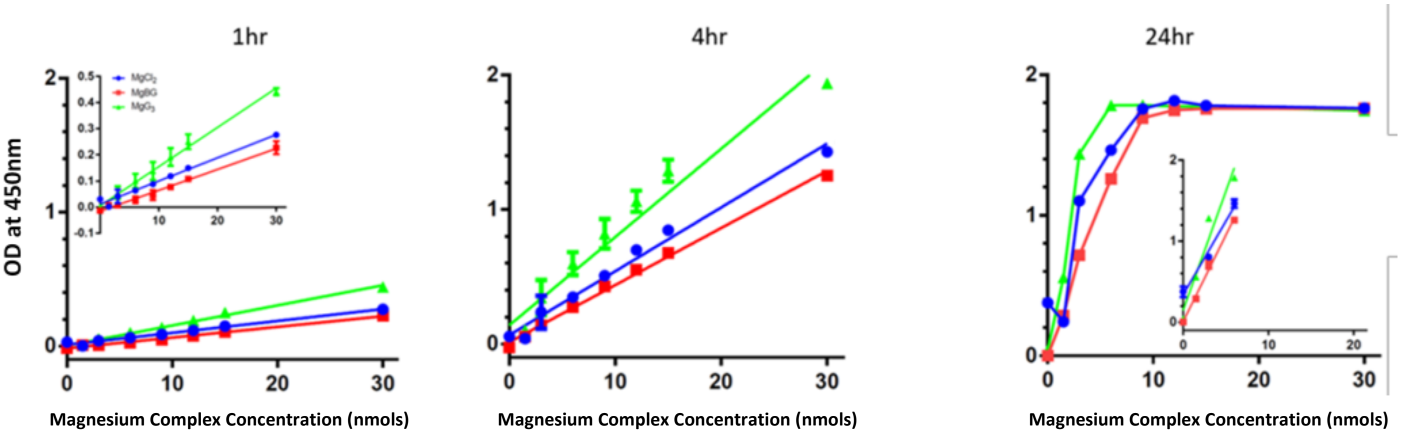

S 18. Kinetic evaluation of the cellular uptake of MgG_3 (**green**), MgBG (**red**), and $MgCl_2$ (**blue**) in CaCo-2 cells analyzed at 1hr (**left**), 4hr (**middle**), and 24hr (**right** – inset included to show kinetic evaluation at points before concentrations that reach cell saturation). Kinetic ratios for MgG_3, MgBG, and $MgCl_2$ are relatively conserved at 1hr and 4hr time points.

S19

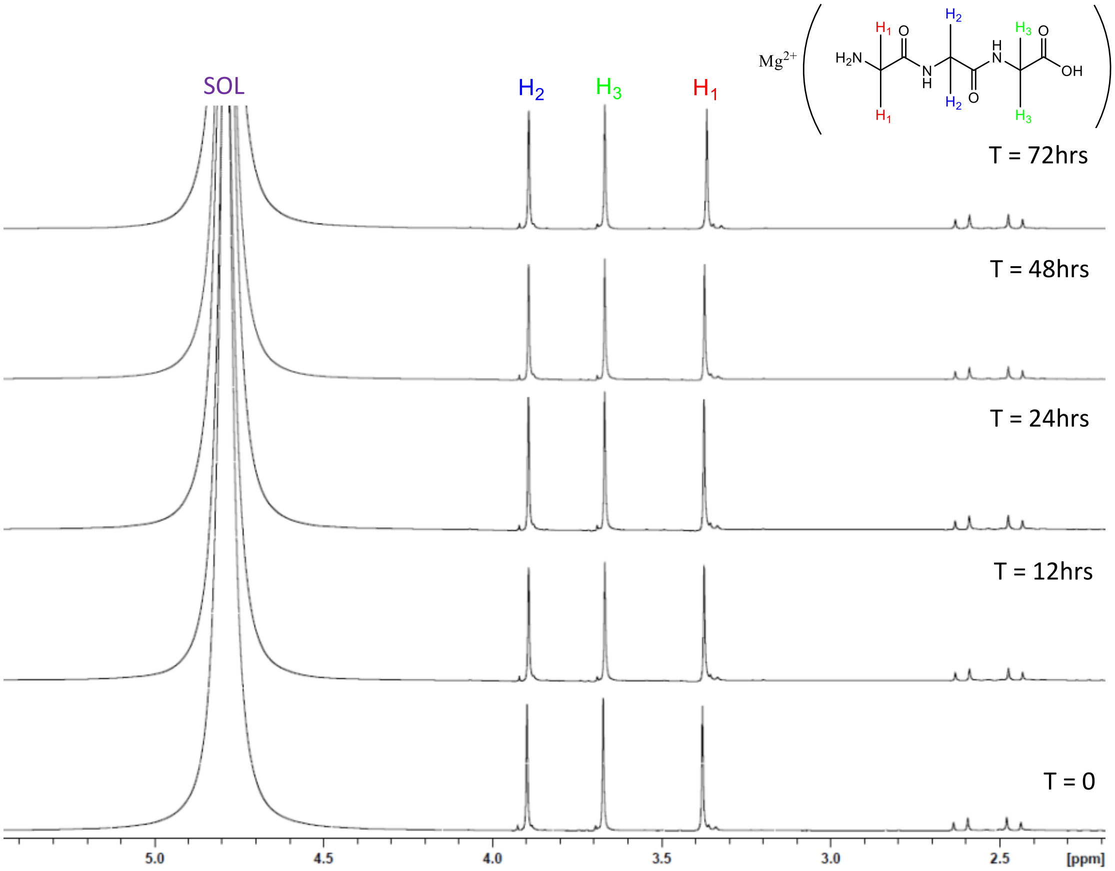

S 19. ^{1}H NMR of MgG_3 in a 1:6 D_2O:H_2O solution showing complex stability over a 72hr period at room temperature.

S20

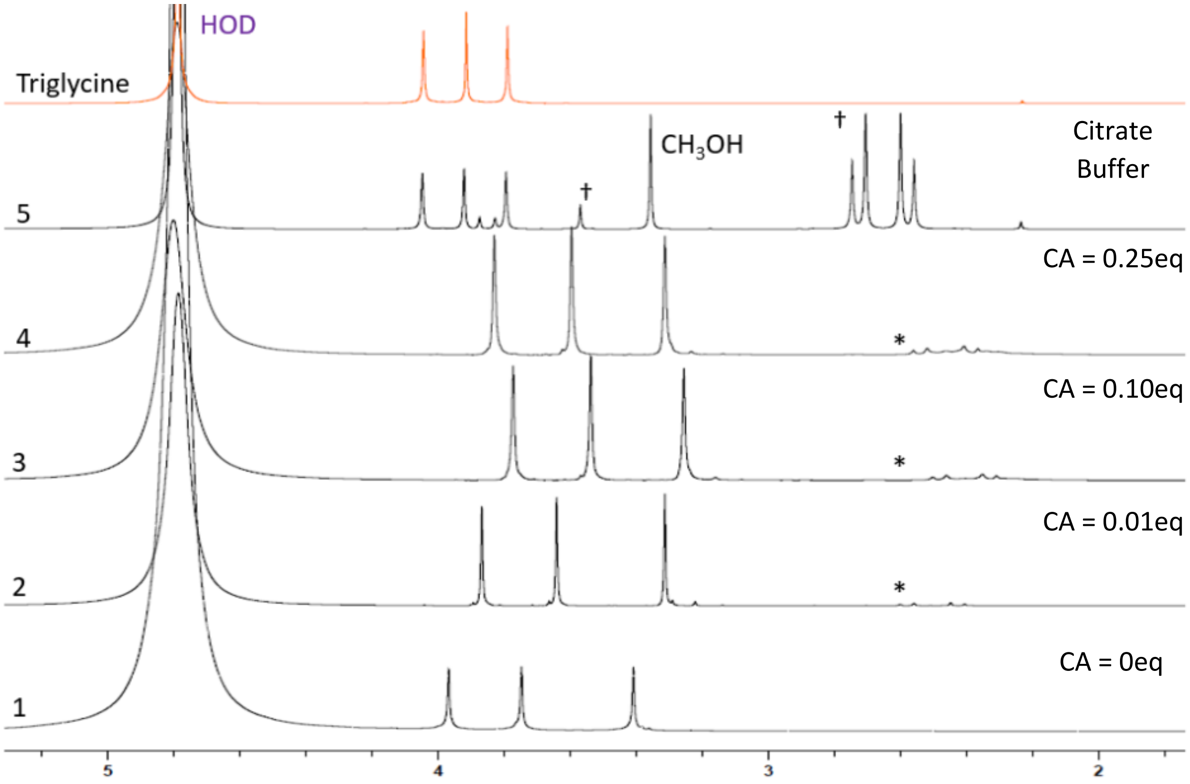

S20. ^{1}H NMR showing the effect of increasing citric acid equivalents on the synthesis of **MgG3**. *Indicates peaks that are attributed solely to citric acid and † indicates peaks that are attributed to magnesium citrate – which are only present in the synthesis conducted in citrate buffer, but not in the synthesis at 0.25eq. of citric acid.

References

(1) Mansour, S. A. A. Thermal Decomposition of Magnesium Citrate 14-Hydrate. *Thermochim. Acta* **1994**, *233* (2), 231–242.

(2) Ding, D.; Lv, L.; Xiao, G.; Ren, Y.; Yang, S.; Yang, P.; Hou, X. One-Step Synthesis of in Situ Multilayer Graphene Containing MgAl2O4 Spinel Composite Powders. *Ceram. Int.* **2019**, *45* (5), 6209–6215.
